# Extensive urogenital calcification and hypercalcemia secondary to disseminated tuberculosis

**DOI:** 10.1590/2175-8239-JBN-2024-0167en

**Published:** 2024-12-13

**Authors:** Aline Grosskopf Monich, Rafael Fernandes Romani

**Affiliations:** 1 Hospital Universitário Evangélico Mackenzie, Curitiba, PR, Brazil.; 2 Faculdade Evangélica Mackenzie do Paraná, Curitiba, PR, Brazil.

Male, 35 years old, drug addict and homeless, admitted with mental confusion, nausea, cough and fever. Laboratory tests showed severe renal dysfunction, hypercalcemia (11.89 mg/dL; RV < 8.8–10.4 mg/dL), and low PTH.

**Figure 1:**
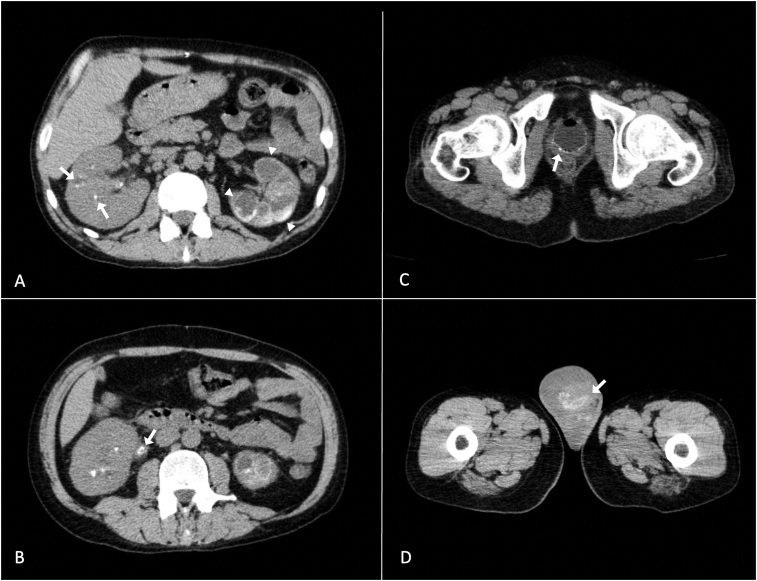
A: calcifications in the right kidney (axial section, arrows) and nephrocalcinosis with caliectasis in the left kidney (axial section, arrowheads); B: ureteral calcification (axial section, arrow); C: contracted and calcified bladder (axial section, arrow); D: hydrocele and testicular calcification (axial section, arrow).

Intermittent hemodialysis was initiated with neurological improvement and a reduction in hypercalcemia. Abdominal CT scan revealed nephrocalcinosis, caliectasis, ureteral and testicular calcifications with contracted and calcified bladder (Figure 1).

The diagnosis was disseminated tuberculosis (TB) with genitourinary involvement, associated with hypercalcemia secondary to granulomatous disease.

The patient was treated for TB, and maintained on an outpatient hemodialysis program due to ESKD. Despite the uncommon manifestations of tuberculosis, the epidemiology and imaging findings led to the final diagnosis.

Genitourinary TB can occur in up to 20% of patients with pulmonary TB and is the second most common site of extrapulmonary TB. Genitourinary involvement is insidious which leads to a diagnostic delay, usually with extensive destruction of the organs involved and symptoms of renal failure. Urinary tract calcification has a low incidence in cases of urogenital TB, and this case demonstrates the alterations typically found^1,2,3^.
