# Supplementary material for: Extensive urogenital calcification and hypercalcemia secondary to disseminated tuberculosis
Source: J Bras Nefrol. 2024 Dec 13;47(1):e20240167. doi: 10.1590/2175-8239-JBN-2024-0167en (PMC11642633; doi:10.1590/2175-8239-JBN-2024-0167en)
Supplement: Translation

# Translation: Calcificação urogenital extensa e hipercalcemia secundárias a tuberculose disseminada

**DOI:** 10.1590/2175-8239-JBN-2024-0167pt

**Authors:** Aline Grosskopf Monich, Rafael Fernandes Romani

**Affiliations:** 1 Hospital Universitário Evangélico Mackenzie, Curitiba, PR, Brasil.; 2 Faculdade Evangélica Mackenzie do Paraná, Curitiba, PR, Brasil.

Homem, 35 anos, dependente químico e morador de rua, admitido com confusão mental, náuseas, tosse e febre. Exames laboratoriais mostraram disfunção renal grave, hipercalcemia (11,89 mg/dL; VR < 8,8–10,4 mg/dL) e PTH baixo.

Iniciada hemodiálise intermitente com melhora neurológica e redução da hipercalcemia. A tomografia de abdome revelou nefrocalcinose, caliectasia, calcificações ureterais e testiculares com bexiga contraída e calcificada (Figura 1).

**Figura 1:** A: focos de calcificação em rim direito (corte axial, setas) e nefrocalcinose com caliectasias em rim esquerdo (corte axial, pontas de setas); B: calcificação ureteral (corte axial, seta); C: bexiga contraída com parede calcificada (corte axial, seta); D: hidrocele e calcificação testicular (corte axial, seta).

O diagnóstico foi tuberculose (TB) disseminada com envolvimento geniturinário, associada à hipercalcemia secundária à doença granulomatosa.

O paciente foi tratado para TB e mantido em programa de hemodiálise ambulatorial devido à DRCT. Apesar de manifestações pouco comuns de tuberculose, a epidemiologia e os achados de imagem levaram ao diagnóstico final.

A TB genitourinária pode ocorrer em até 20% dos pacientes com TB pulmonar e é o segundo sítio de TB extrapulmonar mais comum. O envolvimento genitourinário é insidioso e o diagnóstico é tardio, geralmente já com extensa destruição dos órgãos envolvidos e sintomas de insuficiência renal. A calcificação do trato urinário tem baixa incidência nos casos de TB urogenital, e este caso demonstra as alterações tipicamente encontradas^1,2,3^.
